# Transient and Stable Overexpression of Extracellular Superoxide Dismutase is Positively Associated with the Myogenic Function of Human Skeletal Muscle-Derived Stem/Progenitor Cells

**DOI:** 10.3390/antiox9090817

**Published:** 2020-09-02

**Authors:** Magdalena Nowaczyk, Agnieszka Malcher, Agnieszka Zimna, Wojciech Łabędź, Łukasz Kubaszewski, Katarzyna Fiedorowicz, Kamil Wierzbiński, Natalia Rozwadowska, Maciej Kurpisz

**Affiliations:** 1 Institute of Human Genetics, Polish Academy of Sciences, 60-479 Poznań, Poland; magdalena.przybyl@igcz.poznan.pl (M.N.); agnieszka.malcher@igcz.poznan.pl (A.M.); agnieszka.zimna@igcz.poznan.pl (A.Z.); katarzyna.fiedorowicz@igcz.poznan.pl (K.F.); kamilwierzbinski@outlook.com (K.W.); natalia.rozwadowska@igcz.poznan.pl (N.R.); 2 Department of Orthopedics and Traumatology, W. Dega University Hospital, University of Medical Sciences, 61-545 Poznań, Poland; wlabedz23@gmail.com (W.Ł.); pismiennictwo1@gmail.com (Ł.K.)

**Keywords:** *SOD3*, heart failure, skeletal muscle-derived stem/progenitor cells, myoblasts, apoptosis, oxidative stress, regenerative medicine

## Abstract

In the present study, the genetic modification of human skeletal muscle-derived stem/progenitor cells (SkMDS/PCs) was investigated to identify the optimal protocol for myogenic cell preparation for use in post-infarction heart therapy. We used two types of modifications: *GFP-*transfection (using electroporation) and *SOD3* transduction (using a lentiviral vector). SkMDS/PCs were cultured under different in vitro conditions, including standard (21% oxygen) and hypoxic (3% oxygen), the latter of which corresponded to the prevailing conditions in the post-infarction heart. Transfection/transduction efficacy, skeletal myogenic cell marker expression (CD56), cellular senescence, and apoptosis, as well as the expression of antioxidant (*SOD1*, *SOD2*, and *SOD3*), anti-aging (*SIRT1* and *FOXO*), anti-apoptotic (*BCL2*), and myogenic (*MyoD* and *MyoG*) genes, were evaluated. The percentage of GFP-positive SkMDS/PCs was determined as an indicator of the efficacy of transfection, which reached 55%, while transduction showed better efficiency, reaching approximately 85% as estimated by fluorescence microscopy. The CD56-positive SkMDS/PCs were present in approximately 77% of the tested cells after transient transfection and approximately 96% after transduction. Under standard in vitro culture conditions, the ability of the differentiated, transfected SkMDS/PCs to form myotubes was greater than that of the wild type (WT) cell population (*p* < 0.001), while the cells transduced with the *SOD3* gene exhibited an increase in cell fusion under both standard (*p* < 0.05) and hypoxic conditions (*p* < 0.001). In transduced SkMDS/PCs, we observed a positive influence of *SOD3* overexpression on cell ageing and apoptosis. We observed an increase in the percentage of young cells under standard (*p* < 0.05) and hypoxic (*p* < 0.001) in vitro culture conditions, with a notable decrease in the percentage of senescent and advanced senescent cells in the *SOD3*-overexpressing cell population detected compared to that observed for the untransduced muscle-derived cells. A lower percentage of apoptotic cells was observed for transduced SkMDS/PCs than that for WT cells under hypoxic in vitro culture conditions. In transiently transfected SkMDS/PCs, we observed significantly higher gene expression levels of *SOD2* (almost 40-fold) (*p* < 0.001) and *FOXO* (*p* < 0.05) (approximately 3-fold) under both normoxic and hypoxic culture conditions and of *BCL2* under hypoxia compared to those observed in untreated cells (WT). In addition, myogenic genes showed a significant increase in *MyoD* (almost 18-fold) expression under standard culture conditions (*p* < 0.0001) and decreased *MyoG* expression (approximately 2-fold) after transfection (*p* < 0.05) compared with that detected in the WT skeletal muscle-derived cell control. Taken together, these results demonstrate that *SOD3-*tranduced skeletal muscle-derived cells may have potential for use in the regenerative treatment of the post-infarction heart.

## 1. Introduction

Most reactive oxygen species are generated as by-products during mitochondrial electron transport. Due to the presence of unpaired electrons in separate orbits in its outer electron shell, atomic oxygen can readily form radicals. The sequential reduction of oxygen leads to the formation of a number of reactive oxygen species (ROS), including superoxide, hydrogen peroxide, hydroxyl radical, hydroxyl ion, and nitric oxide [1]. At low concentrations, ROS serve as signaling molecules [2]. The shift in the balance of oxidants and antioxidants in favor of oxidants may lead to phenomenon called “oxidative stress”. ROS are highly reactive molecules that can damage and alter the functions of cell structures, such as carbohydrates, nucleic acids, lipids, and proteins. The regulation of reducing and oxidizing (redox) potential is crucial for cell viability, activation, and proliferation, and for organ function. Aerobic organisms form integrated antioxidant systems comprising enzymatic and non-enzymatic antioxidants that are typically effective in blocking the harmful effects of ROS [3,4].

Living cells have both enzymatic and non-enzymatic defense mechanisms to balance a multitude of oxidative challenges. The enzymatic mechanisms include superoxide dismutase (SOD), catalase, and glutathione peroxidase (GSHPx) [5,6]. Dismutation of O_2_^−^ by SOD results in the generation of H_2_O_2_, which can be further metabolized by catalase into water and oxygen. The non-enzymatic mechanisms include a variety of biological molecules, including, among others, vitamins E and C. In the normal myocardium, as in the other tissues, antioxidants protect cells by maintaining O_2_^−^ and H_2_O_2_ at low levels [7].

Following acute myocardial infarction (MI), oxidative stress develops in both the infarcted and remaining non-infarcted myocardium. Nicotinamide adenine dinucleotide phosphate oxidase (NADPH) is a major source of O_2_^−^ in the heart. After MI, NADPH oxidase expression is significantly increased in the myocardium [8], with neutrophils and macrophages being the primary cells expressing this enzyme. Furthermore, macrophage-derived inducible nitric oxide synthase, a major source of NO during tissue repair, is significantly increased in the infarcted myocardium [9]. As a result, ROS production is elevated in the infarcted myocardium and contributes to the development of oxidative stress in the infarcted heart.

Among the enzymatic defense mechanisms against ROS, extracellular superoxide dismutase is particularly important. Extracellular superoxide dismutase (EC-SOD) is a key antioxidant enzyme implicated in the regulation of ROS-mediated tissue damage. EC-SOD is present in the extracellular matrix of many tissues and is ideally suited to prevent cell and tissue damage initiated by extracellularly produced ROS. In addition, EC-SOD is likely to play an important role in mediating nitric oxide-induced signaling events, since the reaction of superoxide and nitric oxide can interfere with nitric oxide signaling [10]. The elimination of ROS is important, since the overproduction of ROS, such as superoxide anion, has been associated with the pathogenesis of a variety of diseases, including cardiovascular, neurological, and pulmonary disorders. Antioxidant enzymes are partly responsible for maintaining low levels of these oxygen metabolites in tissues and may play key roles in controlling or preventing these conditions [10,11].

Skeletal muscle-derived stem/progenitor cells have been increasingly used in human regenerative medicine, and the potential of genetically modified SkMDS/PCs for use in rebuilding myocardium, regenerating post infarction scars, and mobilizing natural reservoirs with anti-apoptotic and anti-aging properties has been demonstrated in previous studies [12,13], allowing for the restoration of redox homeostasis [14]. Furthermore, it is possible to modify SkMDS/PCs by overexpressing *SOD3*. Myoblasts harboring the *SOD3* gene can become more resistant to the unfavorable hypoxic conditions prevailing in the post-infarction scar and may be a promising approach to improve the regenerative abilities of SkMDS/PC. We decided to use two methods of *SOD3* overexpression in SkMDS/PCs, namely a transient and stable one (in this paper defined as transduction). Both methods have been successfully carried out in our laboratory. However, in our view, while the transient gene transfection can be sufficient for some in vitro analyses, a stable gene expression could be crucial to invoke the expected effect in situ due to the more resistant environment. So, we have been interested in both phenomena, which could be prospectively applied in pre-clinical studies/scenarios.

The aim of this study was to assess the biological properties, including anti-apoptotic and anti-aging effects, of human SkMDS/PCs cultured in vitro and to improve their function by promoting myotube formation when overexpressing extracellular superoxide dismutase. We examined the potential application of extracellular superoxide dismutase gene expression as a possible factor that could be used in the future to modify human SkMDS/PCs, providing them additional proregenerative abilities for myocardial regeneration.

## 2. Materials and Methods

### 2.1. Human SkMDS/PCs Isolation

Human SkMDS/PCs were isolated from residual muscle tissue fragments after surgery intervention in the abdominal rectus area. For this purpose, approval from the Bioethical Local Committee (Poznan University of Medical Sciences, permission no. 818/13) and written consent from the patients were obtained.

SkMDS/PCs were isolated according to a previously modified technique [15,16]. Briefly, pre-purified and fragmented tissue was enzymatically digested with collagenase type II (Sigma-Aldrich, Saint Louis, MO, USA) for 45 min at 33°C and then filtered through mesh, neutralized with balanced Hanks’ solution, and centrifuged for 10 min at 1200 rpm at room temperature. The cells were then cultured in standard Dulbecco’s modified Eagle’s medium containing 4.5 g/L glucose and supplemented with 20% fetal bovine serum (Lonza Group, Basel, Switzerland), 1% antibiotics (Lonza Group, Basel, Switzerland), 1% ultraglutamine (Lonza Group, Basel, Switzerland), and basic fibroblast growth factor (bFGF) (Sigma-Aldrich, Saint Louis, MO, USA). Tissue culture flasks were coated with gelatine, and the cells were incubated under standard (under an atmosphere with 95% humidity and 5% CO_2_ at 37 °C) or hypoxic (under an atmosphere with 95% humidity and 3% CO_2_ at 37 °C) in vitro culture conditions. After every 2–3 days of cultivation, cell confluence was observed, and digested cell suspensions were transferred to another culture flask coated with gelatine as required.

The medium was then changed every other day, and the cells were passaged using 0.25% trypsin with phosphate buffered saline (PBS) (Lonza Group, Basel, Switzerland). The experimental procedures were performed 72 h after *SOD3* transfection and after 7 days of in vitro cultivation after *SOD3* transduction, when the cell confluence reached approximately 75–90%, which was microscopically assessed.

### 2.2. Hypoxia Optimization

The in vitro culture conditions used to grow human SkMDS/PCs under hypoxia were previously determined [17] by plotting the oxygen concentration curve to compare oxygen levels in muscle-derived cells transplanted into the post-infarcted hearts of SCID mice. The cells were further maintained for 24 h and 1 week at the following different oxygen concentrations: 1%, 2%, 3%, 5%, 7%, 10%, and 15%. Next, the expression of the hypoxia-inducible factor 1-alpha (HIF-1a) was evaluated to establish at which oxygen concentration it exhibited the highest expression in SkMDS/PCs [17]. Based on the results of hypoxia optimization, in the present study, cells were cultured in standard in vitro conditions, which reflect 21% O_2_ concentrate (95% humidity and 5% CO_2_, at 37°C), while hypoxia was stabilized at 3% by using N2.

### 2.3. Vectors for Transient Transfection

Genetic modification of SkMDS/PCs was performed with two different plasmids harboring the *SOD3* and the green fluorescent protein (GFP)-encoding genes. The bicistronic vector pIRES2-EGFP was used to express a gene of interest together with *GFP* and was commercially obtained (Clontech, Mountain View, CA, USA). The *SOD* coding sequence was PCR amplified from cDNA templates obtained from mouse muscle cells using primers designed for *SOD3*, and the CDS sequence was obtained from the NCBI (BLAST) database. To express *SOD3* in mammalian cells, the PCR product was cloned into the pIRES2-EGFP vector using the restriction enzymes *Xho*I and *Eco*RI.

### 2.4. Transient Transfection of Human SkMDS/PCs

Human SkMDS/PCs were transfected with the described vectors by electroporation (Gene Pulser X-Cell Electroporation, System Biorad, CA, USA) using the following optimized conditions: One 15-ms pulse, a wave tension of 160 V, and cuvettes with 2-mm gaps. To generate the expected number of transfected cells, we used 3 × 10^6^ muscle-derived cells resuspended in F10 medium at a final volume of 200 µL, and the vector concentration was established according to its size. Due to the high cell mortality triggered by electroporation, the medium was changed 24 h after electroporation. After 48 h, when the transgene was activated, the cells were collected and cultured for 24 h under different culture conditions (3% oxygen concentration was considered as hypoxia and 21% as the standard in vitro culture condition) to assess and compare their properties under different oxygen concentrations. All experiments conducted on human muscle-derived cells were performed 72 h after electroporation.

### 2.5. Transduction of Human SkMDS/PCs

The *SOD3* gene was overexpressed using the vector pLV[Exp]-SV40promoter > EGFP:T2A:Puro-EV1A > hSOD3[NM_003102.2], which was obtained from Vector Builder. This vector coexpresses *EGFP* and a puromycin resistance from the same SV40 promoter and allows for the visualization and selection of transductants carrying the transgene of interest, respectively. Lentiviral particles were packed using a second-generation packaging system. The plasmids pLV[Exp]-SV40promoter > EGFP:T2A:Puro-EV1A > hSOD3, psPAX and MD2G were mixed (4:3:1), and the transfection of human embryonic kidney 293 cells (HEK 293) (27 × 10^6^) was performed using a calcium phosphate protocol. Pseudoviral particles were collected at 48 and 72 h post transfection, filtered through a 45-µm filter (Merck, Darmstadt, Germany), and then centrifuged using Centrifugal Filter Units (Amicon Ultra-15, Merck Millipore, CA, USA). The aliquots were snap frozen and then stored at -80°C. Viral RNA was isolated using a Qiaamp Viral RNA mini kit (Qiagen, Hilden, Germany) following the manufacturer’s instructions. RT-PCR was performed using a Lenti-X PCR Titration kit (Clontech Mountain View, CA, USA) to determine the viral copy number.

An hour before transduction, SkMDS/PCs were treated with polybrene (5 µg/mL), after which a half-volume of medium supplemented with polybrene (5 µg/mL), FGF and lentiviral particles (pLV-GFP-SOD3 or pLV-GFP) were added, and the mixture was incubated for 24 h. The amount of proviruses was adjusted experimentally. After 24 h, the medium was diluted 2-fold by adding cell culture medium to all culture flasks. At 48 h after transduction, the medium was replaced with fresh medium, and at 72 h post-transduction, selection with the appropriate antibiotics was initiated.

### 2.6. Evaluation of CD56-Positive Cells

Flow cytometry was used to assess the population of human SkMDS/PCs three days after transfection/transduction. The transfected/transduced muscle-derived cells were first evaluated by flow cytometry using an anti-CD56 PC5 conjugate (Beckman Coulter, Brea, USA). Briefly, 2.5 × 10^5^ cells were harvested, centrifuged, and resuspended in 100 μL of PBS supplemented with 2% FBS and 10 μL of antibodies (1:200 dilution). Then, after a 20 min incubation, the cells were centrifuged, resuspended in PBS supplemented with 2% FBS, and further analyzed (Beckman Coulter, Brea, CA, USA).

### 2.7. Potential for Myotube Formation

To estimate the differentiation potential of human SkMDS/PCs, the in vitro cultures were maintained under the regime used in the cell differentiation protocol. The cells were cultured in 6-well plates, and 1 mln of cells was examined. Muscle-derived cells were cultured for 1 week in differentiation medium, which was composed of 1% antibiotics (Lonza Group, Basel, Switzerland), 1% ultraglutamine (Lonza Group, Basel, Switzerland), and 2% horse serum (Sigma-Aldrich, Saint Louis, MO, USA). The fusion index (FI) of all cell populations under study was determined. Seven days after the addition of a horse serum-supplemented medium, the differentiated cells were fixed in a freezing methanol:acetic acid (3:1) solution and stained using a Giemsa solution (Merck, Darmstadt, Germany). The images were taken using a standard light microscope, and all cell nuclei were counted (≥ 450 nuclei). FI was defined as the ratio of the number of nuclei present in the differentiated myotubes (Nd) to the total number of nuclei × 100 (FI = (Nd/(Nd+Nnd)) ×100).

### 2.8. Cell Senescence Assay

The senescence-associated expression of β-galactosidase (SA-β- Gal) was evaluated using a Cell Senescence Detection kit (BioVision, Milpitas, CA, USA) to chemically detect SA-β-Gal activity in cells cultured in vitro. SA-β-Gal is only present in senescent cells and has not been observed in pre-senescent, quiescent, or immortal cells. Human SkMDS/PCs were cultured in 6-well dishes and assayed following the manufacturer’s protocol (BioVision, Milpitas, CA, USA). After an overnight incubation at 37 °C, the cells were washed with water and 1% acid alcohol before being stained with eosin for 5 min for better cell visualization. Then, the SkMDS/PCs were observed and counted under a light microscope (Leica DMi8, Wetzlar, Germany).

### 2.9. Detection of Apoptosis

The SkMDS/PCs were collected after transfection/transduction, and the percentage of apoptotic cells was examined using an Annexin V-FITC kit (Beckman Coulter, Fullerton, CA, USA). The cells were washed twice with cold PBS and then resuspended in a binding buffer at a concentration of 5 × 10^6^ cells/mL. Then, 100 μL of the cell suspension were incubated with 1 μL of Annexin-V-FITC solution and 5 μL of propidium iodide (PI) for 15 min in the dark before being analyzed by flow cytometry (Beckman Coulter, Brea, CA, USA). In this assay, the ability of annexin V to bind phosphatidylserine on the cell surface was assessed, where annexin V is linked to fluorescein (FITC) and labels apoptotic cells. Using this assay, the early apoptotic cells (annexin V+/PI-) and late apoptotic cells (annexin V + /PI +) were enumerated.

### 2.10. Gene Expression Analysis

Total RNA was extracted using an AllPrep DNA/RNA/Protein Mini kit (Qiagen, Hilden, Germany), and any potential genomic DNA contamination was eliminated using a Turbo DNA-free kit (Invitrogen, Carlsbad, CA, USA). The purity of the RNA samples was analyzed using a Nanodrop 2000 spectrophotometer (Thermo Scientific, Waltham, MA, USA), and the quality was assessed on a 1.5% agarose gel. Complementary DNA (cDNA) synthesis was performed using 1.5 µg of total RNA and SuperScript IV Reverse Transcriptase (Invitrogen, Carlsbad, CA, USA). The resulting cDNA was diluted 4× and evaluated by PCR with primers for the β-actin gene. Real-time PCR analyses were performed using iQ™ SYBR^®^ Green Supermix (Bio Rad, Warsaw, PL). Each reaction had a final volume of 12.5 μL and contained 2 μL of cDNA, 6.25 μL of 2 × iQ™ SYBR^®^ Green Supermix Reagent (BioRad, Hercules, CA, USA), and 1.25 μL of each primer (4 μM). The following thermocycling conditions were used: 95 °C for 1 min followed by 45 cycles of 95 °C for 20 s, 60 °C for 20 s, and 72 °C for 20 s. *ACT*, *GAPDH*, and *HPRT* were used as reference genes, and the primers used for real-time PCR are presented in Table 1.

### 2.11. Reactive Oxygen Species (ROS) Test

The development of reactive oxygen species (ROS) in the tested cell populations was detected using a DCFDA Cellular ROS Detection Assay Kit (Abcam, Cambridge, UK) according to the producer’s manual. Investigated cells were seeded at a density of approximately 6000 cells per well on a 96-well microplate (flat and clear bottom, dark side), with 10 wells per group. The microplate was read using an up-read plate reader GLOMAX^®^ Multi Detection System (Promega, Madison, WI, USA) with excitation/emission wavelengths filter: 490/510–570 nm. The relative fluorescence of each tested SkMDS/PCs sample was normalized with reference to the fluorescence of wild type myoblast without any reagent.

## 3. Results

### 3.1. Transfection Efficiency

**Figure 1:**
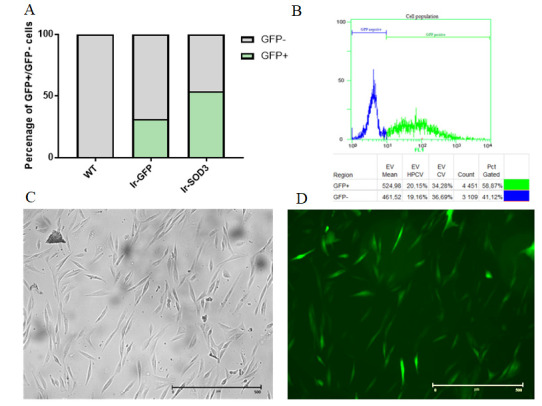
Percentage of positive and negative green fluorescent protein (GFP)-expressing human SkMDS/PC populations after transfection (**A**,**B**). The following cell populations were observed: wild type (WT), untreated myoblasts; vector pIRES2 (Ir), myoblasts transiently transfected with the Ires vector containing either the *GFP* or *GFP-SOD3* sequences. Human SkMDS/PCs transduced/transfected with *SOD3* and were cultured in vitro before being analyzed under a light microscope (**C**) or a fluorescence microscope (**D**) to detect GFP-positive signals.

Transfection efficiency was examined by flow cytometry (Beckman Coulter, Brea, CA, USA). The percentage of GFP-positive cells containing the transgene was determined at 488 nm using a GFP FITC-A detector, with 10,000 events analyzed per sample. Despite using the identical procedure to transfect human SkMDS/PCs, a greater transfection efficiency was observed for the *SOD3* gene than the GFP control (~58 and ~30%, respectively), which was likely due to differences in the size of the plasmids (Figure 1).

### 3.2. Transduction Efficiency

**Figure 2:**
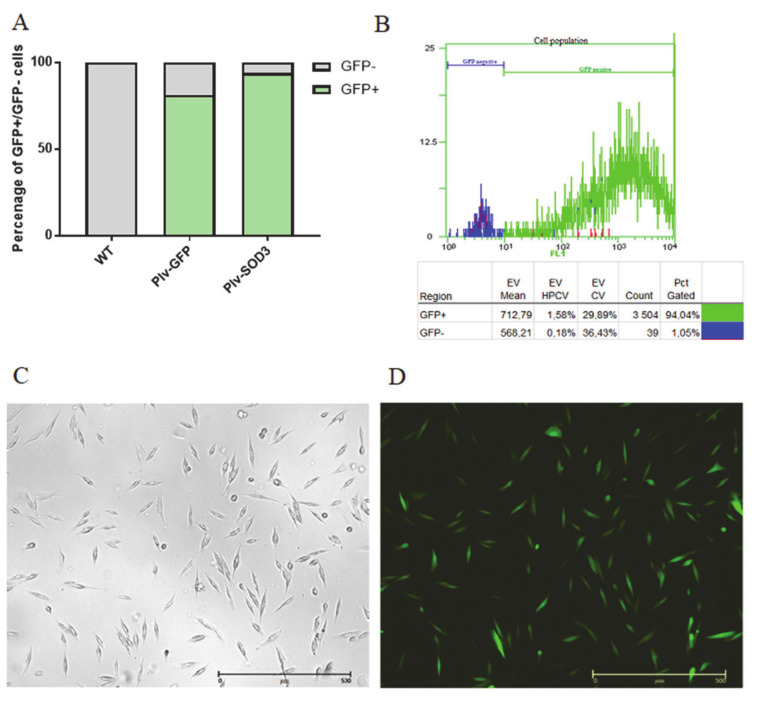
Percentage of positive and negative GFP-expressing human SkMDS/PC populations after the performed transduction (**A**,**B**). The following cell populations were observed: WT, untreated myoblasts; vector pLV[Exp]-SV40 promoter (plV), myoblasts stably transduced with the plV vector containing either the *GFP* or *GFP-SOD3* sequences. Microscopy images of human SkMDS/PCs (**C**,**D**). The *SOD3*-transduced human SkMDS/PC population cultured in vitro was analyzed under a light microscope (**C**) or a fluorescence microscope (**D**) to detect GFP-positive signals.

To assess the efficiency of transduction, we used fluorescence microscope (JuLI Br - Live Cell Movie Analyzer). The green fluorescent protein gene present in the plasmid was coexpressed with *SOD3* in transduced human SkMDS/PCs and/or alone in the control group, allowing muscle-derived cells to be visualized. We observed a positive GFP signal 48 h after transduction, with approximately 90% of cells being GFP positive (Figure 2B).

### 3.3. Phenotype of Human SkMDS/PC Samples

Prior to transfection/transduction, human SkMDS/PCs were harvested and then characterized by flow cytometry using an anti-CD56-specific antibody (differentiation marker for myogenic cells).

**Figure 3:**
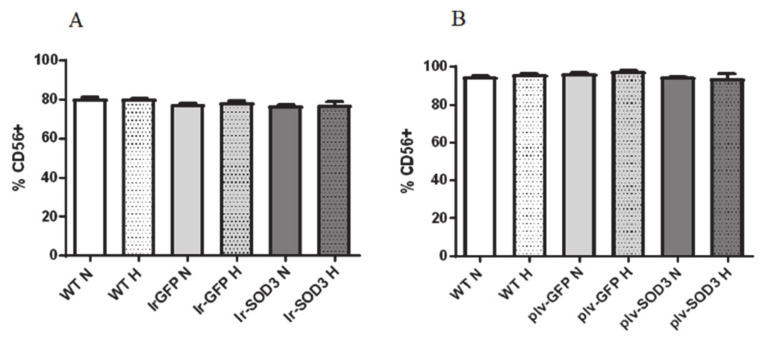
Percentage of differentiation marker for myogenic cells- CD56-positive cells in the in vitro culture of transfected cells (**A**) and transduced human SkMDS/PC population (**B**). The following cell populations were observed: WT, wild type myogenic control population; Ir-GFP, myogenic control population of transient transfection using the Ires-GFP plasmid; plv-GFP, myogenic control population of transduction using the plv-vector; Ir-SOD3, *SOD3*-transfected cell populations; plv-SOD3, *SOD3*-transduced population; cultured under standard (N) and hypoxic (H) conditions.

The flow cytometry results indicated that approximately 77–80% of the transiently transfected cells evaluated in each sample were positive for CD56 (Figure 3).

Next, the results indicated that almost 94–96% of the transduced human SkMDS/PCs evaluated in each analyzed cell sample were positive for CD56. Thus, the transfection and transduction processes did not affect the level of the assayed myoblast marker in the studied cell samples.

### 3.4. Myotube Formation

**Figure 4:**
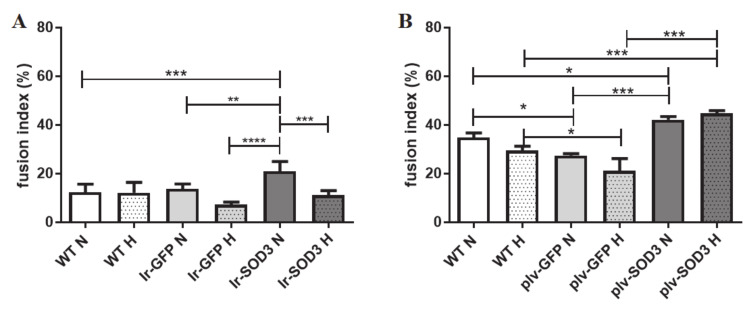
Percentage of fused nuclei in human SkMDS/PC-originating myotubes in the transfected cells cultured in vitro (**A**) and in transduced cell populations (**B**). The following cell populations were observed: WT, wild type muscle-derived cells; Ir-GFP, transiently transfected muscle-derived cells expressing *GFP*; Ir-SOD3, transiently transfected muscle-derived cells expressing *SOD3*; plv-GFP, stably transduced muscle-derived cells expressing *GFP*; plv-SOD3, stably transduced muscle-derived cells expressing *SOD3*; cells cultured under standard (N) and hypoxic (H) conditions.

The population of *SOD3*-transfected/transduced human SkMDS/PCs exhibited a superior ability for myotube formation, as shown in Figure 4.

The human SkMDS/PCs transfected with the *SOD3* gene overexpression construct generated almost 2-fold more fused nuclei than that observed in the WT population under standard in vitro conditions (*p* < 0.001). In contrast, under hypoxic conditions, we did not observe any differences between the studied cell populations (Figure 4A).

The fusion index, a ratio of the number of nuclei in fused cells to the number of unfused cells (100×), under standard and hypoxic conditions was as follows: 12.4/12.5% in the WT control cell population, 13.8/7.3% in the *GFP*-transfected cell population, and 21.1/11.2% in the muscle-derived cells transfected with *SOD3*. Under hypoxic conditions, we noted a 2-fold decrease in the myotube formation potential for the *SOD3*-transfected cells (Figure 4; Table S1). The transduction of human SkMDS/PCs with *SOD3* generated a 10–15% higher rate (*p* < 0.05) of fused nuclei than that observed in the untreated population when cultured under standard and hypoxic conditions (Figure 4B). Thus, the transduction procedure produced a 2-fold greater improvement in cell fusion ability compared to the transfection protocol (Figure 4; Table S1).

### 3.5. Senescence Analysis Based on senescence-associated β-galactosidase (SA-β-gal) Activity

**Figure 5:**
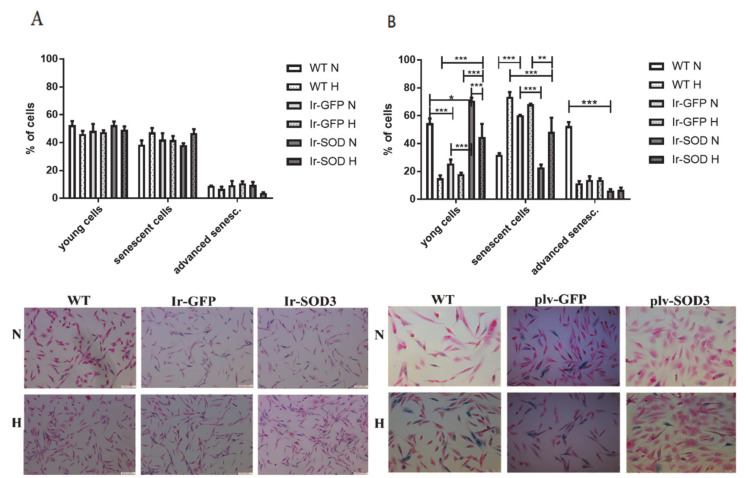
Percentage of young, senescent, and advanced senescent cells in the WT, *GFP*, and *SOD3* overexpressed human Sk MDS/PC populations cultured in standard (N) and hypoxic conditions (H). Analyzed samples included transiently transfected muscle-derived cells (**A**) and transduced muscle-derived cells (**B**). WT—wild type muscle-derived cells, Ir-GFP—transiently transfected muscle-derived cells with *GFP*, Ir-SOD3—transiently transfected muscle-derived cells with *SOD3*, plv-GFP—stable transduced muscle-derived cells with *GFP,* plv-SOD3—stable transducted muscle-derived cells with *SOD3;* N—in standard (nomoxic) and H—hypoxic conditions.

Senescent cells were positively stained for SA-β-gal (Figure 5; Table S2). The percentages of SA-β-gal-positive cells were markedly different under the standard and hypoxic in vitro culture conditions. With respect to transient transfection, the introduction of the *SOD3* gene itself did not contribute to significant changes in the ageing process of the cell population (Figure 5A).

As expected, a high proportion of the young human SkMDS/PCs (SA-β-gal-negative cells) was observed in the *SOD3*-transduced cell population. We observed almost 20% more young cells in the *SOD3*-transduced human SkMDS/PCs compared to that detected in the WT control cell population under standard in vitro culture conditions (*p* < 0.05) and almost 3-fold more young cells under hypoxic conditions (*p* < 0.001). Consequently, we observed almost 25% and 45% fewer senescent human SkMDS/PCs than WT control cells under hypoxic (*p* < 0.001) and standard (normoxia) (*p* < 0.001) (Figure 5B) in vitro culture conditions, respectively, when analyzing the transduced population of human SkMDS/PCs. Even more striking were the reduced populations of advanced senescent human SkMDS/PCs in the transduced cell populations (Figure 5B) (*p* < 0.001).

### 3.6. Apoptosis

**Figure 6:**
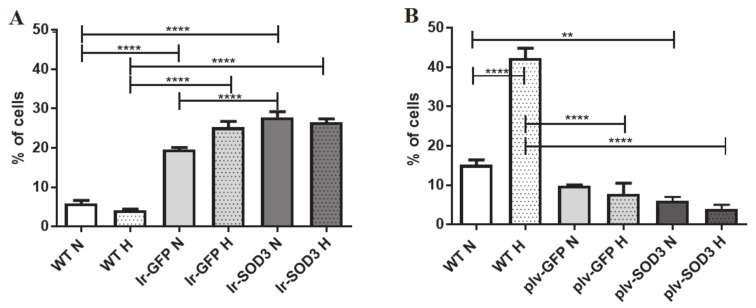
Cell apoptotic rate in the human SkMDS/PC populations under study: WT, Ir-GFP, and Ir-SOD3 are the transfected cells cultured under standard and hypoxic conditions in vitro (**A**), and WT, plV-GFP, and plV-SOD3 are the transduced cells cultured under standard and hypoxic conditions in vitro (**B**). The following cell populations were observed: WT, wild type muscle-derived cells; Ir-GFP, transiently transfected muscle-derived cells expressing *GFP*; Ir-SOD3, transiently transfected muscle-derived cells expressing *SOD3*; plv-GFP; stably transduced muscle-derived cells expressing *GFP*; plv-SOD3, stably transduced muscle-derived cells expressing *SOD3*; cells cultured under (standard (N) and hypoxic (H) conditions.

We detected significantly higher levels of cell mortality in the transiently transfected human SkMDS/PCs than in the WT control cell population, as shown in Figure 6 (*p* < 0.0001) (Table S3).

However, the percentage of apoptotic human SkMDS/PCs was almost 10% and 40% lower under normoxic (*p* < 0.001) and hypoxic (*p* < 0.0001) culture conditions, respectively, compared with that observed in the untreated muscle-derived cell population (WT). These results emphasized that transduction was a significantly better procedure than the transient approach with respect to the rates at which apoptotic cells were obtained.

### 3.7. Gene Expression

**Figure 7:**
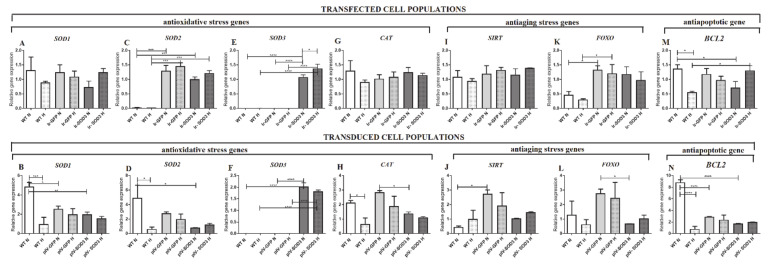
Expression profile of functional genes in human SkMDS/PCs transfected/transduced with *SOD3*, including evaluation of superoxide dismutase 1 (*SOD1*) (**A**,**B**), manganese-dependent superoxide dismutase (*SOD2*) (**C**,**D**), extracellular superoxide dismutase 3 (*SOD3*) (**E**,**F**), catalase (*CAT*) (**G**,**H**), sirtuin 1 (*SIRT1*) (**I**,**J**), forkhead box O3 (*FOXO*) (**K**,**L**), and B-cell lymphoma 2 (*BCL2*) (**M**,**N**). The following cell populations were observed: WT, wild type muscle-derived cells; Ir-GFP, transiently transfected muscle-derived cells expressing *GFP*; Ir-SOD3, transiently transfected muscle-derived cells expressing *SOD3*; plv-GFP, stably transduced muscle-derived cells expressing *GFP*; plv-SOD3, stably transduced muscle-derived cells expressing *SOD3*; cells cultured under standard (N) and hypoxic (H) conditions.

The expression of selected antioxidant and anti-ageing genes was examined in the human SkMDS/PC populations under study. The expression levels of genes coding for dismutase, catalase, sirtuin, *FOXO*, and *BCL2* are shown in Figure 7 (Tables S4 and S5).

We observed no significant differences in the expression level of superoxide dismutase 1 [Cu-Zn] (*SOD1*) between the WT and transiently transfected human SkMDS/PC populations (Figure 7A). However, in both the transfected and transduced populations, a tendency towards increased expression of the *SOD1* gene was observed under hypoxic conditions when compared to the WT human control cells cultured under the same conditions (Figure 7B) (Tables S4 and S5).

Transfection with *SOD3* caused an almost 40-fold increase in the level of manganese-dependent superoxide dismutase 2 (*SOD2*) expression under normoxic (*p* < 0.01) and hypoxic (*p* < 0.001) in vitro culture conditions compared to that observed in the WT control human SkMDS/PC populations (Figure 7C). However, we did not observe a similar effect in the transduced cell population (Figure 7D). We observed significant overexpression of the *SOD3* gene in transfected (*p* < 0.0001) (Figure 7E) and transduced (*p* < 0.0001) muscle-derived cell populations cultured under both standard and hypoxic conditions (Figure 7F, Table S5).

The expression levels of genes encoding catalase (*CAT*) and sirtuin 1 (*SIRT1*) remained at similar levels in all the studied transfected human SkMDS/PC populations under both standard and hypoxic in vitro culture conditions (Figure 7G,I; Table S4). Interestingly, these same genes showed higher levels of expression caused by transduction itself, as shown in (Figure 7H,J; Table S5). We observed similar effects of these procedures (transfection and transduction) on *FOXO* gene expression, although FOXO gene expression had a tendency to be higher in transiently transfected human SkMDS/PCs (Figure 7K,L; Table S5).

The expression level of the anti-apoptotic *BCL2* gene decreased by 50% in muscle-derived human SkMDS/PCs transfected with *SOD3* (*p* < 0.05) and by 5-fold in transduced cells (with *SOD3*) compared to that observed in the WT cell population when cultured under standard conditions (*p* < 0.0001) (Figure 7M,N; Tables S4 and S5). However, a positive effect of *SOD3* gene transfection was observed under hypoxic culture conditions, where *BCL2* gene activity increased more than 2-fold (*p* < 0.05) (Figure 7M; Tables S4 and S5).

**Figure 8:**
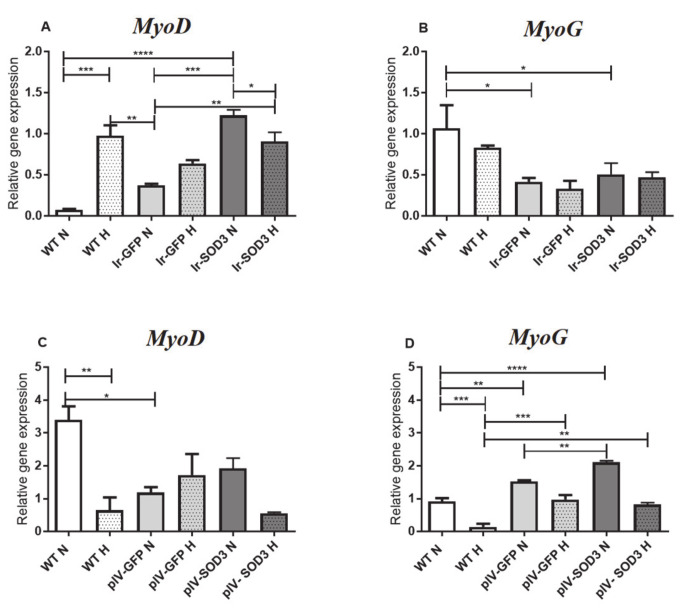
*MyoD* and *MyoG* gene expression in transfected (**A**,**B**) and transduced (**C**,**D**) human SkMDS/PC populations cultured under standard and hypoxic culture conditions in vitro. The following cell populations were observed: WT, wild type muscle-derived cells; Ir-GFP, transiently transfected muscle-derived cells expressing *GFP*; Ir-SOD3, transiently transfected muscle-derived cells expressing *SOD3*; plv-GFP, stably transduced muscle-derived cells expressing *GFP*; plv-SOD3, stably transduced muscle-derived cells expressing *SOD3*; cells cultured under standard (N) and hypoxic (H) conditions.

Transfection with genes encoding superoxide dismutase-3 exerted a positive effect on the studied myogenic genes *MyoD* and *MyoG*. We observed a significant increase in *MyoD* expression under standard in vitro culture conditions for the transfected human SkMDS/PC population compared with that observed in the WT control sample (*p* < 0.0001). In addition, the hypoxic in vitro culture conditions contributed to a significant increase in *MyoD* expression (*p* < 0.001) (Figure 8A). In contrast, we observed a decrease in *MyoG* expression levels (late myogenic factor) after transient human SkMDS/PC transfection compared to that observed in the control cell population (*p* < 0.05) (Figure 8B; Table S4).

Unexpectedly, the expression level of the *MyoD* gene was not increased in the transduced human SkMDS/PC population compared to the WT control cells under either standard or hypoxic conditions (Figure 8C). In contrast, we observed an almost 2-fold increase in *MyoG* expression levels in muscle-derived cells stably transduced with *SOD3* compared to that observed in the WT group under standard in vitro conditions (*p* < 0.0001) and an almost 6-fold increase in *MyoG* expression in *SOD3*-overexpressing muscle-derived cells compared to that observed in the WT group under hypoxic conditions (*p* < 0.01) (Figure 8D; Table S5).

### 3.8. Functional Evaluation of Reactive Oxygen Species (ROS) Activity

**Figure 9:**
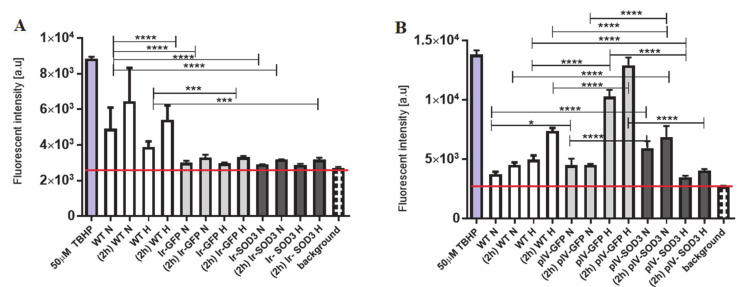
Analysis of the reactive oxygen species activity in the studied populations of human skeletal muscle-derived stem/progenitor cells. The graph illustrates the relative fluorescence in transfected (**A**) and transduced (**B**) SkMDS/PCs compared to WT cells after 45 min of incubation (according to the manufacturer’s protocol) and 2 h of incubation (2 h). Positive control: 50 µM TBHP—myoblasts non-transfected/transduced, treated with 2′,7′-dichlorofluorescin diacetate and 50 µM tert-butyl hydrogen peroxide (TBHP).

The reactive oxygen species activity assay showed that there is a significant difference in ROS activity between WT SkMDS/PCs and cells transfected with *GFP* or *SOD3* after 2 h of incubation with ROS reagent, in the hypoxic condition (*p* < 0.001). After the transduction protocol, we observed significantly higher levels of ROS in the WT SkMDS/PCs and GFP control group compared to *SOD3*-transduced SkMDS/PCs in standard and hypoxic conditions (*p* < 0.001). In standard culture conditions, we observed a lower level of ROS in SkMDS/PCs transduced with the *SOD3* gene than in the GFP control population (*p* < 0.001), and higher than in non-treated cells (*p* < 0.001) (Figure 9; Table S6).

## 4. Discussion

Human SkMDS/PCs cultured in vitro from skeletal muscle oligobiopsies are frequently used for stem cell therapies of myogenic origin [18]. Protocols for muscle-derived cell isolation from skeletal muscles biopsies have been previously reported by our group [15]. Other reports indicated the applicability of SkMDS/PCs for cardiovascular indications in preclinical models of proregenerative therapies [19] as well as for sphincter incontinence [20]. However, it has often been observed that rather high numbers of SkMDS/PCs (≥1×10^8^) are required to obtain visible therapeutic effects or to improve the clinical symptoms in patients. Thus, the potential of in vitro-cultured skeletal muscle-derived stem/progenitor cell populations for clinical purposes requires further protocol optimization. Modifications of SkMDS/PC preparation protocols have been reported that claim to generate large numbers of muscle-derived cells with differentiation and fusion potency (myotubes) that could be sufficient to regenerate damaged muscle, including myocardium. The ability to obtain large numbers of cells in *in* *vitro* cultures is challenging, as their differentiation and myotube formation reduces their ability to propagate [18]. Therefore, it is necessary to find a balance with respect to the myogenicity, proliferation, and fusion potential of human SkMDS/PCs.

Another important issue is the redox status that marks the oxygen concentration in situ, which beyond its indispensable role in cell respiration and metabolism plays a central role in multiple of biological processes and may determine heart functioning [21]. Oxygen imbalance due to myocardial infarction requires a number of antioxidative mechanisms to counteract inflammatory actions and support cardiomyocyte function.

Superoxide dismutase (SOD) is reported to be the major enzyme responsible for protecting against free radicals and common pro-oxidants. Unfortunately, the analysis of EC-SOD (SOD3) serum activity did not show any specific differences between patients with coronary artery disease and control subjects [22]. However, it would be interesting to introduce the *SOD3* gene into failing myocardium using SkMDS/PCs that overexpress this gene. There are several genetic modification approaches used in stem cells, such as transient transfection to introduce foreign DNA or RNA into eukaryotic cells and/or transduction-transfection procedures that use bacteriophages and other vectors [23]. We were the first to undertake genetic modifications of human SkMDS/PCs using two transfection routes in parallel to assess their effects on cells cultured in vitro. Interestingly, compared to transient transfection through electroporation, a greater efficiency of lentiviral transfection (called transduction to distinguish between these two procedures) was demonstrated by observations of GFP-positive cells under a microscope.

There was also no information regarding the influence of *SOD3* overexpression on myogenesis, which, to the best of our knowledge, we were the first to evaluate. It was important that *SOD3* transfection did not exert a negative impact on the phenotype (CD56) of the SkMDS/PC populations nor affect cell differentiation. The presence of the CD56 marker, which is typical of myoblasts, remained similar after the transfection protocol, with approximately 80% of CD56-positive cells being maintained in the obtained population. An even greater proportion (approximately 96%) of CD56-positive cells was obtained after performing the transduction procedure. Thus, similar levels of the CD56 marker can be observed in all muscle-derived cells samples used in both protocols.

The potential of SkMDS/PCs to form the contractile apparatus in vitro could have a positive effect when creating a functional cellular graft within the post-infarction scar in vivo. In skeletal muscle myotubes, the fusion process affects the generation of elongated fibers with longitudinally oriented nuclei, thereby providing instruction for contractile proteins within specific domains along the fiber. Cell–cell fusion is crucial for the normal development of specific tissues and leads to the formation of functional fibers [24], and yet, the nature and degree of conservation of the underlying molecular components remains largely unknown [25]. The potential for cell differentiation into myotubes was assessed using a so-called fusion index. The results showed that the transfection of cells with the *SOD3* gene led to a significant increase in the cell fusion index under standard in vitro culture conditions (Figure 4), which was also observed via the transduction process. Furthermore, the latter approach led to a significant increase in the cell fusion index under both standard and hypoxic in vitro cell culture conditions. Thus, transduction appears to be more effective, because the fusion index was 2-fold higher than that observed in control muscle-derived cells when creating mature muscle fibers.

The relationship between cell ageing and *SOD3* gene expression was first characterized in lung disorders with respect to fibroproliferation and excessive deposition of extracellular matrix in late adulthood. The exact mechanisms linking ageing and fibroproliferative disorders have remained unknown, but increased oxidative stress resulting in the accumulation of damaged DNA, lipids, and proteins can be considered to be a major possible factor [26]. In the lung, and especially in the pulmonary fibroblasts, extracellular superoxide dismutase (EC-SOD) is a major antioxidant enzyme, and indirect modification of the expression of this factor has been performed. It was subsequently observed that old fibroblasts exhibited only marginally elevated levels of reactive oxygen species (ROS), which coincided with the attenuated expression of a number of antioxidant enzymes, including EC-SOD, while the restoration of *SOD3* gene expression could prevent the excessive deposition of extracellular matrix in late adulthood. Thus, pathological manifestations become less prominent in adult individuals [26]. We confirmed the effect of *SOD3* overexpression on the ageing process of skeletal muscle-derived cells in vitro using a common test that takes advantage of cell senescence being associated with β-galactosidase activity (SA-β-Gal). Thus, we showed a positive influence of *SOD3* transduction on SkMDS/PCs, showing a high percentage of young cells and low percentages of senescent and advanced senescent skeletal muscle-derived cells (significant) when compared to untreated samples under standard and hypoxic in vitro cell culture conditions. Moreover, an even more significant effect (*p* < 0.001) was observed under hypoxic conditions in in vitro cell cultures (Figure 5B). A similar effect was not observed after the transfection procedure, which may be related to an insufficient amount of time of gene exposure (*SOD3*) due to the transient transfection protocol.

Similar results were also observed with respect to apoptosis. In our study, we observed significant differences between the levels of apoptosis in the WT control muscle-derived cells and the SkMDS/PCs transduced with the *SOD3* gene (Figure 6). Apoptosis was decreased under both the standard (*p* < 0.01) and hypoxic (*p* < 0.001) in vitro cell culture conditions for SkMDS/PCs stably overexpressing the *SOD3* gene. In the untransduced control population (WT), we observed a large influence of the hypoxic conditions on the increase in apoptosis. The effect of EC-SOD on the apoptotic process was also marked in a mouse model in which human recombinant protein (hEC-SOD) was introduced through intraperitoneal injection. The protective effects of hEC-SOD were attributed to an enhanced nuclear translocation of nuclear factor E2-related factor 2 (Nrf2), which subsequently increased the expression of NAD(P)H dehydrogenase 1 and heme oxygenase-1. Consequently, the application of hEC-SOD resulted in the recovery of animals from systemic and renal inflammation and apoptosis, as reflected by decreased monocyte chemoattractant protein-1 and tumor necrosis factor-α levels in serum and an increased B-cell lymphoma 2 (BCL-2)/ Bcl-2-associated X-protein (BAX) ratio in diabetic kidneys [27].

The positive effect of *SOD3* overexpression on myotube formation, cell ageing, and apoptosis prompted us to examine changes in the molecular background of SkMDS/PCs functional genes. To this end, we assessed the expression of selected antioxidant (*SOD1* and *SOD2*), anti-aging (*SIRT* and *FOXO*), and anti-apoptotic genes (*BCL2*). We observed positive and significant changes in *SOD2* gene expression under standard and hypoxic culture conditions after *SOD3* transfection compared to that observed in untreated cells (WT), which clearly demonstrates the biological effect of *SOD3* overexpression. The transcriptional regulation of all three superoxide dismutase isoforms is tightly controlled based on extra- and intracellular redox conditions [28]. *SOD1* and *SOD2* mRNA levels are increased in response to a wide array of mechanical, chemical, and biological messengers, such as heat shock [29], shear stress [30], or hydrogen peroxide [29] produced by extracellular superoxide dismutase (*SOD3*). Because catalase eliminates H_2_O_2_ from the cell environment, which is a byproduct of a reaction catalyzed by *SOD3*, the increased expression of catalase may be expected [31]. However, in our observations, *CAT* expression levels in transfected human SkMDS/PCs were similar to those in all transfected skeletal muscle-derived cell variants and were rather reduced in *SOD3-*transduced muscle-derived cells (Figure 7G,H). The reason for this phenomenon observed in the in vitro culture may be an overexpressed catalase buffer capacity as well as a lack of an extracellular environment capable of scavenging free radicals, which is quite possible when the cells act under in vivo conditions.

Higher levels of *SOD2* and *FOXO* gene expression were observed after human SkMDS/PCs transfection and for *CAT*, *FOXO*, and *SIRT1* gene expression after SkMDS/PCs transduction; unfortunately, this phenomenon was also observed in the *GFP*-transfected controls, albeit at an insignificant level. Along this line, we observed some sensitivity with respect to the upregulation of these genes caused by the protocol itself. In this study, for the first time, we assayed genes that may function in response to changes in the redox environment and mutually correlate with each other. This could occur due to the upregulation of antioxidant genes (*SOD2* and *CAT*) in response to cellular stress or due to applied physical stressors (electroporation) that could induce the transcription of *SOD2* or other factors, including cytokines, during an induced short inflammation period. In our case, the stress was induced through mechanical electroporation (transfection) and strong viral transduction. The external stressing factor (transfection/transduction procedure) can also be responsible for the observed increase in anti-aging gene expression (*FOXO* and *SIRT*) (Figure 8I,J).

In addition, we studied the expression of myogenic transcription factors under various in vitro culture conditions (standard ones and hypoxic). High expression of pro-myogenic genes can indicate a specific predisposition of the cells for prospective myogenesis. Myogenic transcription factors are expressed in a specific sequence during myoblast activation. We examined the transcription factor *MyoD*, which is typically expressed at earlier stages than myogenin (*MyoG*), which is considered to be a late myogenic factor [32]. This sequential expression in different stages of myogenesis plays a crucial role in skeletal muscle development, e.g., *MyoD* plays a role in muscle cell specialization while myogenin controls the differentiation process and is associated with the formation of myotubes [33]. In the human SkMDS/PC population transiently transfected with *SOD3*, we observed a significant increase (*p* < 0.0001) in *MyoD* expression compared to that detected in the WT cell population in standard conditions. At the same time, we showed the impact of hypoxia alone on the increased expression of the early myogenic factor (Figure 8A). In turn, the transfection-mediated overexpression of *SOD3* clearly caused a decrease in *MyoG* transcription (Figure 8A). Both of these results indicate a positive effect of *SOD3*-overexpressing SkMDS/PCs in maintaining the myogenicity and proliferation potential of genetically modified SkMDS/PCs. Thus, we can conclude that the transfection process may maintain the proliferative potential of the muscle-derived cells transiently overexpressing *SOD3*, which could be relevant when planning to use these muscle-derived cells for treatment of the post-infarcted heart. A tendency towards increased *MyoD* expression was also observed in *SOD3-*transduced cells, although we observed a rather significant increase in *MyoG* expression under both standard and hypoxic in vitro cell culture conditions (Figure 8B). It may be that high levels of *MyoG* can cause the cytoplasmic retention of this protein through a mechanism that regulates the biological activity of this transcription factor. Myogenin can be retained in the cytoplasm to prevent cell differentiation until the proper mitogenic signaling. After translocation into the nucleus, myogenin exerts transcriptional activity on specific myogenic promoters, thereby leading to complete muscle differentiation, which is correlated with the predominant localization of myogenin in myotube nuclei [34].

The results of the expression of antioxidant genes, showing a positive effect of *SOD3* overexpression on other genes, such as catalase, SOD1, or SOD2, but ambiguous in comparison with the control GFP group, prompted us to perform a functional test for free radicals relieving modulation by the SkMDS/PC populations. The results of the dichlorodihydrofluorescein diacetate (DCFDA) assay in the *SOD3*-transfected population showed lower levels of free radicals: hydroxyl, peroxyl, and other reactive oxygen species within the cell in comparison with the WT group only in the case of a longer incubation time with ROS regent in both standard (*p* < 0.001) and hypoxic (*p* < 0.01) conditions. In the case of SkMDS/PCs transduction, we observed a positive effect of *SOD3* overexpression on the presence of free radicals in hypoxia. There were significantly fewer free radicals than in the control (GFP) and not treated (WT) group (*p* < 0.001).

Whether this specific mechanism may play a positive role in *SOD3*-transduced human SkMDS/PCs is unknown at present. However, transduction of the *SOD3* gene is undoubtedly beneficial for myogenic function (myotube formation), with excellent viability observed under hypoxic conditions. Therefore, enhanced *SOD3* gene activity may promote better human SkMDS/PCs proregenerative properties when promptly delivered to the post-infarction site.

## 5. Conclusions

Introduction of *SOD3* to SkMDS/PCs was studied in respect to both transient and stable processes of gene overexpression in human SkMDS/PCs both in standard as well as hypoxic cell in vitro culture conditions. The functional capacity of myotube formation was maintained after positive *SOD3* overexpression. In transduced SkMDS/PCs, a positive (and significant) trend for cell anti-senescence and anti-apoptosis was continued, although the gene expression concerning other antioxidant and/or anti-aging genes was selective. An important hint was observed concerning early transcription factor gene expression (*MyoD*), which was even more pronounced in transiently transfected SkMDCS/PCs. Although the final change in human myogenic cells was not fully consistent, the benefits of *SOD3* of such genetically modified cells in prospective cellular techniques are evident.

## Figures and Tables

**Table 1:** Primers sequences and expected length of the PCR product.

	Sequence (5′- > 3′)	Length of the PCR Product
***CAT***		
Forward primer	TATCCTGACACTCACCGCCA	277
Reverse primer	CGTTCACATAGAATGCCCGC	
***BCl2***		
Forward primer	GGATAACGGAGGCTGGGATG	123
Reverse primer	TATTTGTTTGGGGCAGGCAT	
***FOXO1***		
Forward primer	GAGGGTTAGTGAGCAGGTTAC	243
Reverse primer	TGGCACAGTCCTTATCTACAG	
***SIRT1***		
Forward primer	TGGTATTTATGCTCGCCTTGC	220
Reverse primer	CAGCGTGTCTATGTTCTGGGT	
***SOD1***		
Forward primer	TGGTTTGCGTCGTAGTCTCC	168
Reverse primer	GTCCATTACTTTCCTTCTGCTC	
***SOD2***		
Forward primer	ACCTGCCCTACGACTACGG	262
Reverse primer	AACTCCCCTTTGGGTTCTCC	
***SOD3***		
Forward primer	ATGCTGGCGCTACTGTGTTC	100
Reverse primer	ACTCCGCCGAGTCAGAGTT	
***MyoD***		
Forward primer	ACGGCATGATGGACTACAG	212
Reverse primer	CGACTCAGAAGGCACGTC	
***MyoG***		
Forward primer	GCTGTATGAGACATCCCCCTA	226
Reverse primer	CGACTTCCTCTTACACACCTT	

## Supplementary Materials

The following are available online at https://www.mdpi.com/2076-3921/9/9/817/s1, Table S1: Statistical differences between the cell fusion index presented in Figure 4, Table S2: Statistical differences between percentage of young, senescent and advanced senescent myogenic cells in the WT, *GFP* and *SOD3* overexpressed human SkMDS/PC populations presented in Figure 5, Table S3: Statistical differences between cell apoptotic rates in the human SkMDS/PC populations of senescent and advanced senescent cells in the WT, *GFP* and *SOD3* overexpressed human SkMDS/PC populations presented in Figure 6, Table S4: Statistical differences between gene expression profile of functional genes in human SkMDS/PCs transfected with *SOD3* presented in Figure 7 and Figure 8, Table S5: Statistical differences between gene expression profile of functional genes in human SkMDS/PCs transducted with *SOD3* presented in Figure 7 and Figure 8, Table S6: Statistical differences between ROS activity in human SkMDS/PCs population under study presented in Figure 9.
