# Supplementary material for: Transient and Stable Overexpression of Extracellular Superoxide Dismutase is Positively Associated with the Myogenic Function of Human Skeletal Muscle-Derived Stem/Progenitor Cells
Source: Antioxidants (Basel). 2020 Sep 2;9(9):817. doi: 10.3390/antiox9090817 (PMC7555722; doi:10.3390/antiox9090817)
Supplement: Supplementary file 1 [file antioxidants-09-00817-s001.pdf]

Table 1. Statistical differences between the cell fusion index presented in Figure 4.

1 way ANOVA test	FUSION INDEX-statistical significance	
	TRANSIENT *SOD3* OVEREXPRESSION (TRANSFECTION)	STABLE *SOD3* OVEREXPRESSION (TRANSDUCTION)
WT N vs WT H	ns	ns
WT N vs Ir/plv-GFP N	ns	*
WT N vs Ir/plv-GFP H	ns	***
WT N vs Ir/plv-SOD N	**	*
WT N vs Ir/plv-SOD H	ns	**
WT H vs Ir/plv-GFP N	ns	ns
WT H vs Ir/plv-GFP H	ns	*
WT H vs Ir/plv-SOD N	**	ns
WT H vs Ir/plv-SOD H	ns	***
Ir/plv-GFP N vs Ir/plv-GFP H	ns	ns
Ir/plv-GFP N vs Ir/plv-SOD N	**	***
Ir/plv-GFP N vs Ir/plv-SOD H	ns	ns
Ir/plv-GFP H vs Ir/plv-SOD N	****	ns
Ir/plv-GFP H vs Ir/plv-SOD H	ns	***
Ir/plv-SOD N vs Ir/plv-SOD H	***	ns

Table 2. Statistical differences between percentage of young, senescent and advanced senescent myogenic cells in the WT, *GFP* and *SOD3* overexpressed human SkMDS/PC populations presented in Figure 5.

2 way ANOVA test	Cell senescence -statistical significance					
	TRANSIENT *SOD3* OVEREXPRESSION (TRANSFECTION)			STABLE *SOD3* OVEREXPRESSION (TRANSDUCTION)		
	Young cells	Senescent cells	Advanced senescent cells	Young cells	Senescent cells	Advanced senescent cells
WT N vs WT H	ns	ns	ns	*	***	***
WT N vs Ir/plv-GFP N	ns	ns	ns	***	***	***
WT N vs Ir/plv-GFP H	ns	ns	ns	***	***	***
WT N vs Ir/plv-SOD N	ns	ns	ns	*	ns	***
WT N vs Ir/plv-SOD H	ns	ns	ns	ns	ns	***
WT H vs Ir/plv-GFP N	ns	ns	ns	ns	ns	ns
WT H vs Ir/plv-GFP H	ns	ns	ns	ns	ns	ns
WT H vs Ir/plv-SOD N	ns	ns	ns	***	ns	***
WT H vs Ir/plv-SOD H	ns	ns	ns	***	***	ns
Ir/plv-GFP N vs Ir/plv-GFP H	ns	ns	ns	ns	ns	ns
Ir/plv-GFP N vs Ir/plv-SOD N	ns	ns	ns	***	***	ns
Ir/plv-GFP N vs Ir/plv-SOD H	ns	ns	ns	ns	ns	ns
Ir/plv-GFP H vs Ir/plv-SOD N	ns	ns	ns	ns	**	ns
Ir/plv-GFP H vs Ir/plv-SOD H	ns	ns	ns	***	ns	ns
Ir/plv-SOD N vs Ir/plv-SOD H	ns	ns	ns	***	ns	ns

Table S3. Statistical differences between cell apoptotic rates in the human SkMDS/PC populations of senescent and advanced senescent cells in the WT, *GFP* and *SOD3* overexpressed human SkMDS/PC populations presented in Figure 6

1 way ANOVA test	Apoptosis-statistical significance	
	TRANSIENT *SOD3* OVEREXPRESSION (TRANSFECTION)	STABLE *SOD3* OVEREXPRESSION (TRANSDUCTION)
WT N vs WT H	ns	****
WT N vs Ir/plv-GFP N	****	ns
WT N vs Ir/plv-GFP H	****	ns
WT N vs Ir/plv-SOD N	****	**
WT N vs Ir/plv-SOD H	****	**
WT H vs Ir/plv-GFP N	****	****
WT H vs Ir/plv-GFP H	****	****
WT H vs Ir/plv-SOD N	****	****

WT H vs Ir/plv-SOD H	****	****
Ir/plv-GFP N vs Ir/plv-GFP H	***	ns
Ir/plv-GFP N vs Ir/plv-SOD N	****	ns
Ir/plv-GFP N vs Ir/plv-SOD H	****	ns
Ir/plv-GFP H vs Ir/plv-SOD N	ns	ns
Ir/plv-GFP H vs Ir/plv-SOD H	ns	ns
Ir/plv-SOD N vs Ir/plv-SOD H	ns	ns

Table S4. Statistical differences between gene expression profile of functional genes in human SkMDS/PCs transfected with *SOD3* presented in Figure 7-8

1 way ANOVA test	Gene expression								
	TRANSIENT *SOD3* OVEREXPRESSION (TRANSFECTION)								
	SOD1	*SOD2*	*SOD3*	*CAT*	*SIRT*	*FOXO*	*BCL2*	*MyoD*	*MyoG*
WT N vs WT H	ns	ns	ns	ns	ns	ns	*	***	ns
WT N vs Ir/plv-GFP N	ns	***	ns	ns	ns	*	ns	ns	*
WT N vs Ir/plv-GFP H	ns	***	ns	ns	ns	ns	ns	**	*
WT N vs Ir/plv-SOD N	ns	**	****	ns	ns	ns	*	****	*
WT N vs Ir/plv-SOD H	ns	***	****	ns	ns	ns	ns	***	*
WT H vs Ir/plv-GFP N	ns	***	ns	ns	ns	*	*	**	ns
WT H vs Ir/plv-GFP H	ns	***	ns	ns	ns	*	ns	*	ns
WT H vs Ir/plv-SOD N	ns	**	****	ns	ns	*	ns	ns	ns
WT H vs Ir/plv-SOD H	ns	***	****	ns	ns	ns	*	ns	ns
Ir/plv-GFP N vs Ir/plv-GFP H	ns	ns	ns	ns	ns	ns	ns	ns	ns
Ir/plv-GFP N vs Ir/plv-SOD N	ns	ns	****	ns	ns	ns	ns	***	ns
Ir/plv-GFP N vs Ir/plv-SOD H	ns	ns	****	ns	ns	ns	ns	**	ns
Ir/plv-GFP H vs Ir/plv-SOD N	ns	ns	****	ns	ns	ns	ns	**	ns
Ir/plv-GFP H vs Ir/plv-SOD H	ns	ns	****	ns	ns	ns	ns	ns	ns
Ir/plv-SOD N vs Ir/plv-SOD H	ns	ns	*	ns	ns	ns	ns	*	ns

Table S5. Statistical differences between gene expression profile of functional genes in human SkMDS/PCs transducted with *SOD3* presented in Figure 7-8

1 way ANOVA test	Gene expression								
	STABLE *SOD3* OVEREXPRESSION (TRANSDUCTION)								
	SOD1	*SOD2*	*SOD3*	*CAT*	*SIRT*	*FOXO*	*BCL2*	*MyoD*	*MyoG*
WT N vs WT H	***	*	ns	*	ns	ns	****	**	***
WT N vs Ir/plv-GFP N	*	ns	ns	ns	*	ns	****	*	**
WT N vs Ir/plv-GFP H	**	ns	ns	ns	ns	ns	****	ns	ns
WT N vs Ir/plv-SOD N	**	*	****	ns	ns	ns	****	ns	****
WT N vs Ir/plv-SOD H	**	*	****	ns	ns	ns	****	**	ns
WT H vs Ir/plv-GFP N	ns	ns	ns	**	ns	ns	*	ns	****
WT H vs Ir/plv-GFP H	ns	ns	ns	ns	ns	ns	ns	ns	***
WT H vs Ir/plv-SOD N	ns	ns	****	ns	ns	ns	ns	ns	****
WT H vs Ir/plv-SOD H	ns	ns	****	ns	ns	ns	ns	ns	**
Ir/plv-GFP N vs Ir/plv-GFP H	ns	ns	ns	ns	ns	ns	ns	ns	**
Ir/plv-GFP N vs Ir/plv-SOD N	ns	ns	****	*	ns	ns	ns	ns	**
Ir/plv-GFP N vs Ir/plv-SOD H	ns	ns	****	*	ns	ns	ns	ns	**
Ir/plv-GFP H vs Ir/plv-SOD N	ns	ns	****	ns	ns	ns	ns	ns	****
Ir/plv-GFP H vs Ir/plv-SOD H	ns	ns	****	ns	ns	ns	ns	ns	ns
Ir/plv-SOD N vs Ir/plv-SOD H	ns	ns	ns	ns	ns	ns	ns	ns	****

Table S6. Statistical differences between ROS activity in human SkMDS/PCs population under study presented in Figure 9

1 way ANOVA test	ROS activity -statistical significance	
	TRANSIENT *SOD3* OVEREXPRESSION (TRANSFECTION)	STABLE *SOD3* OVEREXPRESSION (TRANSDUCTION)
50mM TBHP vs. WT N	****	****
50mM TBHP vs. (2h) WT N	****	****

50mM TBHP vs. WT H	****	****
50mM TBHP vs. (2h) WT H	****	****
50mM TBHP vs. Ir/plV-GFP N	****	****
50mM TBHP vs. (2h) Ir/plV-GFP N	****	****
50mM TBHP vs. Ir/plV-GFP H	****	****
50mM TBHP vs. (2h) Ir/plV-GFP H	****	ns
50mM TBHP vs. Ir/plV-SOD3 N	****	****
50mM TBHP vs. (2h) Ir/plV-SOD3 N	****	****
50mM TBHP vs. Ir/plV- SOD3 H	****	****
50mM TBHP vs. (2h) Ir/plV- SOD3 H	****	****
50mM TBHP vs. background	****	****
WT N vs. (2h) WT N	**	*
WT N vs. WT H	ns	****
WT N vs. (2h) WT H	ns	****
WT N vs. Ir/plV-GFP N	****	*
WT N vs. (2h) Ir/plV-GFP N	*	ns
WT N vs. Ir/plV-GFP H	****	****
WT N vs. (2h) Ir/plV-GFP H	**	****
WT N vs. Ir/plV-SOD3 N	****	****
WT N vs. (2h) Ir/plV-SOD3 N	**	****
WT N vs. Ir/plV- SOD3 H	****	ns
WT N vs. (2h) Ir/plV- SOD3 H	**	ns
WT N vs. background	****	****
(2h) WT N vs. WT H	****	ns
(2h) WT N vs. (2h) WT H	ns	****
(2h) WT N vs. Ir/plV-GFP N	****	ns
(2h) WT N vs. (2h) Ir/plV-GFP N	****	ns
(2h) WT N vs. Ir/plV-GFP H	****	****
(2h) WT N vs. (2h) Ir/plV-GFP H	****	****
(2h) WT N vs. Ir/plV-SOD3 N	****	****
(2h) WT N vs. (2h) Ir/plV-SOD3 N	****	****
(2h) WT N vs. Ir/plV- SOD3 H	****	***
(2h) WT N vs. (2h) Ir/plV- SOD3 H	****	ns
(2h) WT N vs. background	****	****
WT H vs. (2h) WT H	**	****
WT H vs. Ir/plV-GFP N	ns	ns
WT H vs. (2h) Ir/plV-GFP N	ns	ns
WT H vs. Ir/plV-GFP H	ns	****
WT H vs. (2h) Ir/plV-GFP H	ns	****
WT H vs. Ir/plV-SOD3 N	ns	***
WT H vs. (2h) Ir/plV-SOD3 N	ns	****
WT H vs. Ir/plV- SOD3 H	ns	****
WT H vs. (2h) Ir/plV- SOD3 H	ns	*
WT H vs. background	*	****
(2h) WT H vs. Ir/plV-GFP N	****	****
(2h) WT H vs. (2h) Ir/plV-GFP N	**	****
(2h) WT H vs. Ir/plV-GFP H	****	****
(2h) WT H vs. (2h) Ir/plV-GFP H	***	****
(2h) WT H vs. Ir/plV-SOD3 N	****	****
(2h) WT H vs. (2h) Ir/plV-SOD3 N	***	ns
(2h) WT H vs. Ir/plV- SOD3 H	****	****

(2h) WT H vs. (2h) Ir/plV- SOD3 H	***	****
(2h) WT H vs. background	****	****
Ir/plV-GFP N vs. (2h) Ir/plV-GFP N	ns	ns
Ir/plV-GFP N vs. Ir/plV-GFP H	ns	****
Ir/plV-GFP N vs. (2h) Ir/plV-GFP H	ns	****
Ir/plV-GFP N vs. Ir/plV-SOD3 N	ns	****
Ir/plV-GFP N vs. (2h) Ir/plV-SOD3 N	ns	****
Ir/plV-GFP N vs. Ir/plV- SOD3 H	ns	***
Ir/plV-GFP N vs. (2h) Ir/plV- SOD3 H	ns	ns
Ir/plV-GFP N vs. background	ns	****
(2h) Ir/plV-GFP N vs. Ir/plV-GFP H	ns	****
(2h) Ir/plV-GFP N vs. (2h) Ir/plV-GFP H	ns	****
(2h) Ir/plV-GFP N vs. Ir/plV-SOD3 N	ns	***
(2h) Ir/plV-GFP N vs. (2h) Ir/plV-SOD3 N	ns	****
(2h) Ir/plV-GFP N vs. Ir/plV- SOD3 H	ns	*
(2h) Ir/plV-GFP N vs. (2h) Ir/plV- SOD3 H	ns	ns
(2h) Ir/plV-GFP N vs. background	ns	****
Ir/plV-GFP H vs. (2h) Ir/plV-GFP H	ns	****
Ir/plV-GFP H vs. Ir/plV-SOD3 N	ns	****
Ir/plV-GFP H vs. (2h) Ir/plV-SOD3 N	ns	****
Ir/plV-GFP H vs. Ir/plV- SOD3 H	ns	****
Ir/plV-GFP H vs. (2h) Ir/plV- SOD3 H	ns	****
Ir/plV-GFP H vs. background	ns	****
(2h) Ir/plV-GFP H vs. Ir/plV-SOD3 N	ns	****
(2h) Ir/plV-GFP H vs. (2h) Ir/plV-SOD3 N	ns	****
(2h) Ir/plV-GFP H vs. Ir/plV- SOD3 H	ns	****
(2h) Ir/plV-GFP H vs. (2h) Ir/plV- SOD3 H	ns	****
(2h) Ir/plV-GFP H vs. background	ns	****
Ir/plV-SOD3 N vs. (2h) Ir/plV-SOD3 N	ns	*
Ir/plV-SOD3 N vs. Ir/plV- SOD3 H	ns	****
Ir/plV-SOD3 N vs. (2h) Ir/plV- SOD3 H	ns	****
Ir/plV-SOD3 N vs. background	ns	****
(2h) Ir/plV-SOD3 N vs. Ir/plV- SOD3 H	ns	****
(2h) Ir/plV-SOD3 N vs. (2h) Ir/plV- SOD3 H	ns	****
(2h) Ir/plV-SOD3 N vs. background	ns	****
Ir/plV- SOD3 H vs. (2h) Ir/plV- SOD3 H	ns	ns
Ir/plV- SOD3 H vs. background	ns	****
(2h) Ir/plV- SOD3 H vs. background	ns	****
